# Research on the Sustainable Renewal of Architectural Heritage Sites from the Perspective of Extenics—Using the Example of Tulou Renovations in LantianVillage, Longyan City

**DOI:** 10.3390/ijerph20054378

**Published:** 2023-02-28

**Authors:** Xianli You, Yanqin Zhang, Zhigang Tu, Linxin Xu, Liyi Li, Rong Lin, Kaida Chen, Shunhe Chen, Wei Ren

**Affiliations:** 1 College of Landscape Architecture and Art, Fujian Agriculture and Forestry University, Fuzhou 350002, China; 2 Jinshan College of Fujian Agriculture and Forestry University, Fuzhou 350002, China

**Keywords:** extenics, Tulou, Tulou renovation, architectural heritages

## Abstract

Fujian Tulous in China are important international architectural heritage sites that reflect precious human cultural heritage. Currently, only a small number of Tulou buildings have been listed as world cultural heritage sites, resulting in a lack of attention and financial support for most Tulou buildings. Thus, it is difficult to effectively renovate and repair Tulou buildings to adapt to modern life, and therefore they are facing the severe challenge of abandonment and desolation. Due to the special conditions of Tulou buildings, there are significant limitations in renovation and repair work, with a number of problems such as the lack of innovative renovations. Therefore, through a problem model analysis of a design system for Tulou renovations, in this study, we adopt the methods of divergent tree, conjugate pair, correlative net, implied system, and split-merge chain analyses in extenics to carry out extension transformation and solve the problem and we verify its feasibility using the example of the Tulou renovation projects in Lantian Village, Longyan City. We explore an innovative methodology for scientific renovation of Tulou buildings, and we establish a design system for Tulou building renovations that enriches and supplements original renovation methods; thus, we provide a basis for the repair and reuse of Tulou buildings, to extend their service life and to realize the sustainable development of Tulou buildings. The research results show that extenics can be implemented in innovative renovations of Tulou buildings, and it is concluded that the essence of achieving sustainable renewal in Tulou building renovations is to solve contradictory problems, including contradictions in conditions, objectives, and design. This study verifies the possibility of applying extenics in the design of Tulou building renovations, makes corresponding contributions to the application of extension methods in the renovation and renewal of Tulou buildings, and also contributes to the renovation, renewal, and protection of other types of architectural heritage sites.

## 1. Introduction

Diversified world heritage sites represent unique human achievements and natural wonders, and they are driving forces that promote human economic, social, and environmental development. Thus, reasonable protection policies should be formulated; however, research fields and heritage values vary among heritage systems. Therefore, world heritage sites are facing more extensive and complex protection and management issues [1]. By 2021, it had been 36 years since China joined the UNESCO’s Convention Concerning the Protection of the World Cultural and Natural Heritage. As of July 2021, China had a total of 56 world heritage sites, including 38 cultural heritage sites, 14 natural heritage sites, and 4 mixed cultural and natural heritage sites (dual heritage). The total number of natural heritage sites ranks first in the world. In the past 36 years, China has achieved an important position in the world heritage family.

For UNESCO, the original intention of entitling world cultural heritage sites is to permanently preserve world cultural heritage sites as the shared cultural heritage of all mankind. The importance of culture is the greatest value connotation of world heritage sites, especially exchanges among different cultures [2]. Tulou buildings are an important part of world cultural heritage and a precious source of human cultural heritage. They are also one of the most distinctive architectural heritage sites, and known as “the most special folk house in China” [3]. At the 32nd World Heritage Conference in 2008, the cultural landscape of Fujian Tulou buildings was successfully added to the World Heritage List based on authenticity and completeness, and meeting the requirements of the following relevant articles: Article (ii), at a certain stage or in a certain cultural region of the world, the Tulou serves as the core exchange of mass social ideology, and it has a significant impact on the developments of architecture, art or heritage art, and town planning or landscape design; Article (iii), the Tulou provides distinctive evidence of the civilization and cultural traditions that have been inherited or have long disappeared; Article (iv), the Tulou is an unparalleled demonstration of the construction, physical composition, related technology, and environmental art, and it comprehensively shows the cultural standards of the last or many critical periods in the process of human development [4]. However, currently, only 46 Tulou buildings have been included in the list, and only 3733 Tulou buildings have been officially recognized. There are still a large number of Tulou buildings that have not been officially certified [5]. Although these buildings are of great importance, a large number of valuable Tulou buildings have still been ignored or even abandoned because they have no world heritage status. In addition, the original functions of Tulou buildings are not able to comprehensively support people’s productive and higher quality of life; thus, an increasing number of local residents believe that they should be rebuilt and renovated [6].

**Figure 1:**
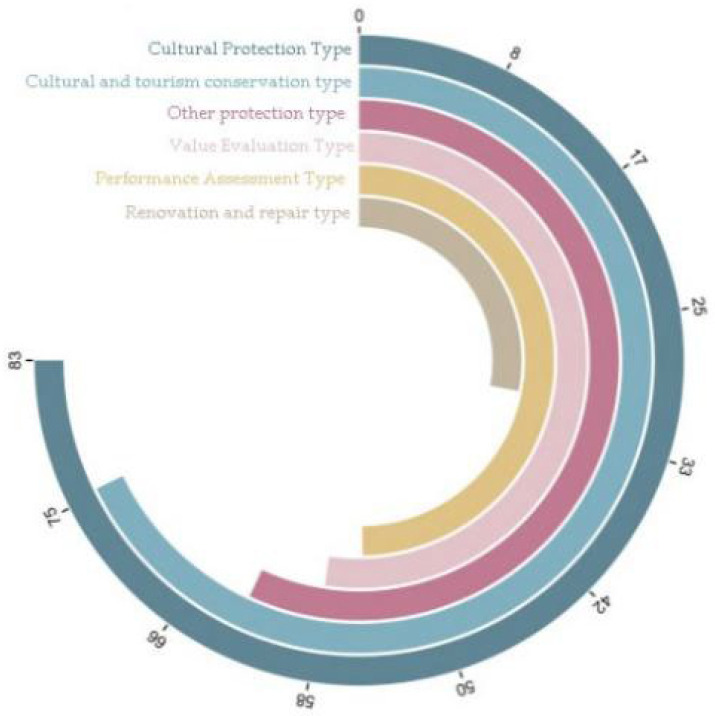
Types of research on Tulou buildings.

**Figure 2:**
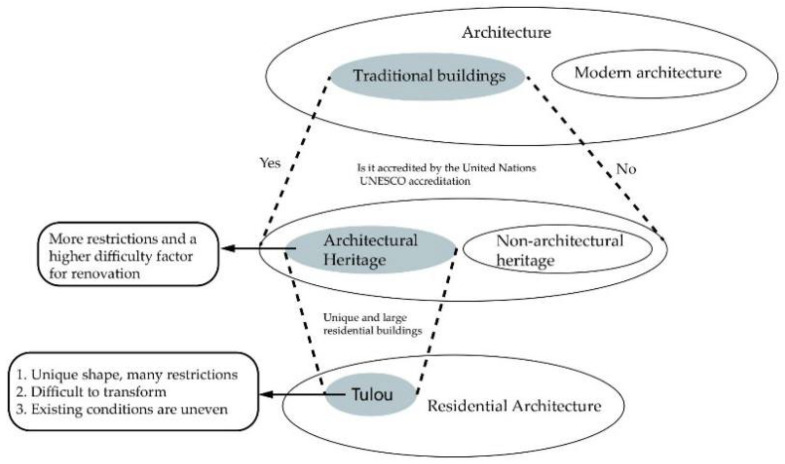
Analysis of the conditions.

Therefore, we conducted in-depth research and reviewed a large number of relevant materials in the literature [7,8,9,10,11,12,13]. Accordingly, the types of research on Tulou buildings can be roughly summarized into six categories: cultural protection, cultural tourism protection, function research, value assessment, renovation and repair, and other protection. The distribution of the different types of research on Tulouo buildings is shown in Figure 1. As shown in the figure, there have been relatively few studies on the renovation and repair of Tulou buildings. The main reasons for this include: (1) Tulou buildings are architectural heritage sites, and therefore there are many restrictions on the renovation, renewal, protection, and repair of these sites. (2) Tulou buildings have significant historical and cultural value with unique internal structures that lead to difficulties in renovations. (3) The existing conditions of most Tulou buildings are varied, and thus there are various problems which are difficult to solve (Figure 2). There are a large number of Tulou buildings that are not only widely distributed but also rich in types; thus, there are limitations in discussing the renovation methods as well as analyzing and solving problems, which has resulted in a large number of contradictory problems that have not been completely solved in the process of renovating Tulou buildings. In addition, Tulou building renovations have the characteristics of comprehensiveness, complexity, and fuzziness, and their design thinking is a black box process. A large number of contradictory problems and ideas may be omitted or deviated in the design process. Studies in the literature have been extensively reviewed [14,15,16,17,18,19,20,21,22] in order to solve this problem, and it has been found that extenics theory has been used to solve complex contradictory problems and to effectively solve various problems existing in the design of Tulou building renovations. This provides a formal expression basis for the renovation of Tulou buildings; thus, the thinking process of renovation design can be communicated and simulated. Thereby, the scientific renovation of Tulou buildings can be realized.

Extenics is a discipline based on formal models for studying the possibility of expanding things and the methods of innovation laws, and it can be used to deal with contradictory problems [22]. Since its establishment, extenics has attracted extensive attention in many research fields. A system framework has been established that mainly consists of a problem model, a problem analysis, and contradictory problem solving, which is frequently used in design fields such as architectural design, landscape design, and product design [17,23,24,25]. Especially in recent years, many scholars have combined extenics with innovative architectural designs to study extenics solutions for the uncertainty of architectural design [24]. Zou, G. T. [16,26] first studied extension architectural design, explored the relationships between extenics and architectures, proposed new disciplines such as “extension architectural design”, and combined it with the protection and design of cultural building relics, which further promoted the application and development of extenics in the field of architecture. Wang, T. et al. [27] combined extenics with the interior theory of architectural space, and put forward a space element and extension model to describe space contradictions. Xue, M. H. further improved the depth of the structure and research content of the extension architectural design system, and verified its rationality and feasibility [28]. Wang, K. Q. et al. [17] constructed the strategy generation mechanism of extension architectural design innovation. Gao, Z. H. [29] summarized the color design matching law of urban residential building facades through extension data mining, and put forward color selection guidance for urban residential building color design. Wang, T. et al. [19] applied extenics to the design of traditional dwelling renovations to explore the generation process of extension strategies for them. Most of these research results have been focused on the design problems of common architectural renovations, but have rarely involved the design and renewal of architectural heritage renovations. Therefore, in this paper, we take the design of Tulou architectural heritage renovations as the research object to explore the application of extenics in the renovation process of Tulou buildings.

To date, few scholars have combined extenics theory with research on the design of Tulou architectural heritage renovations. In the design of Tulou building renovations, taking into consideration the unique conditions of Tulou buildings, design contradictions often occur in the renovation process. Therefore, in this study, we adopt extenics to solve contradictions in the design of Tulou renovations; we explore the application of extenics in Tulou building renovations; we introduce the basic element theory, thinking modes, and transformation methods of extenics in the design of Tulou renovations; and we verify the feasibility of applying extenics in Tulou renovations. Finally, a new design system is developed to express, explain, operate, and apply the design of Tulou building renovations, which provides more methods for designers to solve design problems in the process of Tulou building renovations and reuse. Through the above methods, the design process of Tulou building renovations can be explained in a rigorous structural system and expressed in a formal way. Thus, contradictions at different levels in the design process of Tulou renovations can be understood systematically and completely, which facilitates the solution of problems and provides a more concise and intuitive description for the design of Tulou renovations. The research results aim to provide a relatively scientific and feasible renovation method and to build a reasonable design system for Tulou building renovations; thus, providing a new thinking mode and practical suggestions for the renovation of Tulou buildings and other types of architectural heritage sites, and increasing experience for the reuse and sustainable renewal of architectural heritage sites in the future.

## 2. Materials and Methods

### 2.1. Overview of the Research Area

**Figure 3:**
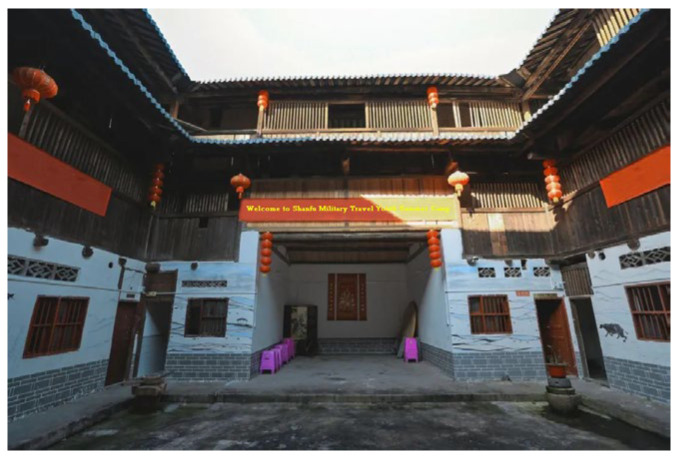
The Yude Building that has been transformed into a summer camp site.

**Figure 4:**
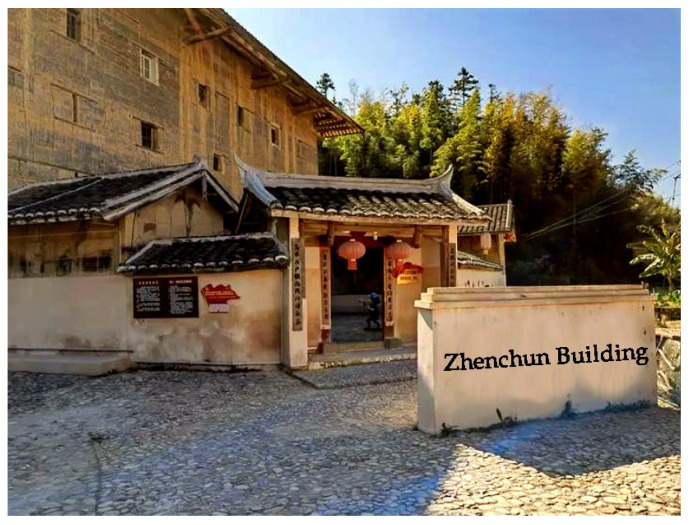
The Zhenchun Building.

The Yude Building and the Zhenchun Building are two Tulou buildings located in Lantian Village, Shizhong Town, Longyan City in Fujian Province. With a history of about 100 years, they are typical architectural heritage sites and are also among the large number of non-world-heritage Tulou buildings. In recent years, the Yude Building has been transformed by the villagers into a summer camp school site due to various reasons such as underdeveloped equipment (Figure 3). However, the layout of the Tulou building has not changed much, and only bathrooms and shower rooms have been added behind the Tulou building. The Zhenchun Building is the nearest Tulou building to the entrance of Lantian Village, with a history of more than 300 years. It is the first square Tulou building with four floors. Despite a history of hundreds of years, the Zhenchun Building is still structurally complete and magnificent. It can still be called the “King of Tulou Buildings” in the village (Figure 4).

The Yude Building and the Zhenchun Building have not yet been certified as world cultural heritage sites, and therefore they have received less attention from outside sources. In addition, transportation between Lantian Village and the urban area is quite convenient, and a large number of young and middle-aged people in the village have left their hometown to work outside the village, resulting in a significant loss of residents in the Tulou buildings, which has changed the inherent human–land dependency of traditional villages [9]. Due to underdeveloped facilities, empty buildings, etc., the Yude Building and the Zhenchun Building have been idle and abandoned. In this circumstance, Lin, J. H., an associate professor at the School of Architecture of the University of Hong Kong who focuses on the transformation of rural architecture and sustainable rural development and is one of the founders of the Rural Urban Framework, led students and craftsmen to carefully investigate and analyze these two Tulou buildings in the summer of 2019. Since these two buildings are not listed as world heritage sites, there are fewer restrictions on their repair work, and they can be flexibly renovated to meet the different functional needs of modern life and to accommodate a series of facilities and users. Therefore, Lin, J. H., who has been working on the renovation of rural buildings in China for more than ten years and has extensive experience in the renovation of rural buildings (he has won many architectural design awards such as for the Angdong Hospital in Hunan and The Pinch project in Yunnan) [30,31,32,33,34], proposed a creative plan, breaking the conventional thinking mode of Tulou building repairs. He believes that protecting a Tulou is not just to protect one thing, but to renovate it so that it can come alive and it can link its history to the present. While not making specific changes to the living environment of Tulou buildings, they used the “acupuncture” method to add an “artistic installation” of wood components in each Tulou building, using minimal effort and considering greater effect: A “funnel staircase” was added in the front yard of the Yude Building to achieve the effect of connecting a community again and a “high tower” was established in the atrium of the Zhenchun Building so that the space could be felt from a different angle without touching any old structure [35]. Meanwhile, the specific details of the renovation of Yude Building and Zhenchun Building can be learned through the following website: https://www.archdaily.cn/cn/940296/ji-ti-sheng-huo-de-zai-si-cheng-cun-jia-gou-xiang-gang-da-xue (accessed on 25 January 2022).

### 2.2. Research Methods

In extenics, three basic elements, namely matter element (M), affair element (A), and relation element (R), are used to describe the design elements, which are usually a set of subinformation from one dimension to multiple dimensions (see Table 1). The one-dimensional formula is expressed as B = (O_m_, C_m_, V_m_), where B represents the basic elements, O_m_ is the object, Cm is the name of the characteristics, and V_m_ is the value of C_m_. Then, multidimensional elements can be represented by:

(1) $$B-|=\left[\begin{array}{ccccc} O_{m} & , & C_{m1} & , & V_{m1}\\ & & C_{m2} & & V_{m2}\\ & & \ldots & , & \ldots \\ & & C_{mn} & & V_{mn} \end{array}\right].$$

In this paper, on the basis of the primitive elements, the basic elements are expanded by using divergent tree, conjugate pair, correlative net, implied system, and split-merge chain analyses to solve a contradictory problem, and these analysis methods are described as follows:

(1) Divergent tree analysis

A divergent tree analysis focuses on the dispersion of objects, features, and quantitative values of primitives. Through the dispersion analysis of objects, the internal structure of things and their basic components are reflected in order to capture the innovative elements for solving contradictory problems. “One object with multiple signs, one action with multiple signs, one sign with multiple objects, one sign with multiple values” constitute the common principles of divergence [36]. For example, if the basic elements are analyzed by divergence and expanded into multiple same-object basic elements, the divergence tree can be expressed as follows:

(2) $$R=\left(O,\;c,\;v\right)-|\left\{\left(O,\;c_{1},\;v_{1}\right),\;\left(O,\;c_{2},\;v_{2}\right),\;\cdots ,\;\left(O,\;c_{n},\;v_{n}\right)\right\}$$

(2) Conjugate pair analysis

The conjugate pair mode of thinking is a unique mode of extenics thinking that mainly starts from the materiality, systemic, dynamic, and oppositional nature of things, and accordingly, completely describes the structure and composition of things with four conjugate pairs: virtual and real, soft and hard, potential and obvious, positive and negative. Then, it converts different conjugate pairs to each other under certain conditions [37], in order to study how to solve problems and achieve the desired goals. Conjugate pair thinking can be represented by a model as follows:

(3) $$O_{m}=\mathrm{Virtual}+\mathrm{Real}=re\left(O_{m}\right)+im\left(O_{m}\right)\;\;=\mathrm{Soft}+\mathrm{Hard}=sf\left(O_{m}\right)+hr\left(O_{m}\right)\;\;\;\;\;\;\;\;\;=\mathrm{Potential}+\mathrm{Obvious}=it\left(O_{m}\right)+ap\left(O_{m}\right)\;\;\;\;\;\;\;\;\;\;\;\;=\mathrm{Positive}+\mathrm{Negative}=psc\left(O_{m}\right)+ngc\left(O_{m}\right)$$

(3) Correlative net analysis

**Figure 5:**
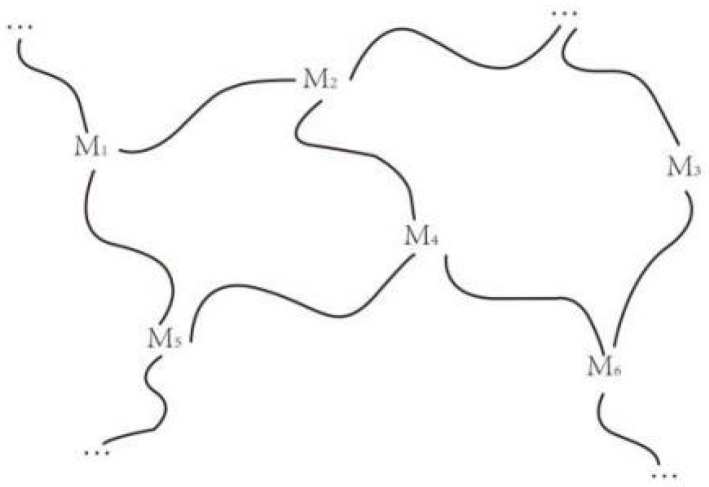
Correlative net.

In extenics, the correlative net analyzes the factors related to things formally and quantitatively to find ways to solve contradictory problems. When a contradictory problem cannot be effectively solved, the correlations between matter elements can be considered, and other matter elements related to it can be adopted to study [38]. There are connections among many things in the world. When the value of one of the characteristics of a matter element changes, the value of its related characteristics will also change. The interconnectedness of matter elements is represented as a mesh structure in a graphical pattern (as shown in Figure 5, with the symbol “~” indicating “correlation”), i.e., M1~M2 means M1 is associated with M2 (Figure 5).

(4) Implied system analysis

**Figure 6:**
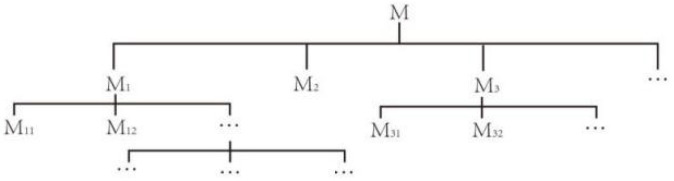
Implied system analysis.

Implied system principles can be adopted to analyze the objectives or conditions of a contradictory problem. By conducting an implied system analysis, as well as analyzing and considering problems at the next level, we can solve problems at the previous level. According to the definition of basic elements implication in the literature [39], it is assumed that the realization of the basic elements of M1 must have the realization of the basic elements of M2, which is called M1 implication M2 and can be expressed as M1⇒M2, where the basic elements on the left side of “⇒” are the lower basic elements and the upper basic elements are on the right side, i.e., M1 represents the lower basic elements and M2 represents the upper basic elements. The realization of basic elements can be written as “basic-elements@”, i.e., the realization of the basic elements of M1 can be written as “M1@”. The implication system is an implication system consisting of multiple upper basic elements together with lower basic elements, as shown in Figure 6.

(5) Split-merge chain analysis

Everything can be combined and decomposed to form new things. The split-merge chain analysis combines and decomposes elements according to the additivity (+), integrability (×) and separability (−) [40] of things to find ways to solve contradictory problems. For given basic elements M1 and M2, M1 and M2 can be added or multiplied, namely M = M1 + M2 or M = M1 × M2. However, when M is composed of many elements that are added together, under certain conditions, it can be decomposed into several elements, which can be expressed as M = (O, C, V)//{M1, M2, M3… Mn}. This new thing that evolves from the combination and decomposition may have some characteristics that the original thing does not possess, which provides the possibility for innovative design.

## 3. Extenics Analysis of Tulou Renovation Design

In the field of architectural design, extenics usually transforms contradictory problems into compatible problems by changing the conditions and objectives of the problems to remove contradictions. The essence of extenics is to solve contradictory problems in architectural design. Through a review of the literature [16,17,19,24,25,26,27,29,41,42], we found that an extension analysis is one of the main tools for architectural design innovation, and also one of the main ways to provide innovative thinking. In addition, extenics is an innovative mode of thinking to improve design efficiency in disciplines such as landscape design. For example, the design of Longmen Square in Acheng City, Heilongjiang Province, is a typical case of creative works produced by using extenics thinking [22], where the designer used extenics thinking for the analysis, through the expansion of a research object, to achieve a clearer design goal, to avoid losing direction due to the complex conditions of the current situation, and then to coordinate the conditions of all parties to promote the design of Longmen Square. Thus, the designer provided a fresh approach to the traditional way of design thinking. As compared with the traditional way of design thinking, using extenics thinking to standardize the design process of symbolic expression significantly shortens the time to organize ideas. In addition, it can be targeted to the complex classification and organization of current conditions, with a shorter time to find the contradictory problem. Thus, it strengthens the design, feasibility, and scientific integrity; therefore, the innovative ideas are clearer and, to a certain extent, the efficiency of the design process is improved, and thus a plan is conceived in a shorter time period. However, the design of Longmen Square only uses a dispersion tree analysis method, a single method with certain limitations, no application of the practical strategies proposed, and weaker communication with the public. Hence, the extension methods can also be applied to the innovation of Tulou buildings. Through a comprehensive analysis of the divergence and conjugation of a Tulou building, the extension transformation of conditions, needs, and goals is carried out, and a variety of analysis methods in the theory of extenics are integrated in order to propose a design system for Tulou renovations that combines the opinions of multiple groups and reduces the occurrence of contradictions; therefore, it can address the needs of modernization while protecting key characteristic elements. In this way, a Tulou building can be renovated or repaired without damaging its historical significance and value, providing a reference for the protection and renovation of Tulou buildings.

In the summer of 2019, associate professor Lin, J. H., at the School of Architecture at the University of Hong Kong, led students and craftsmen to conduct a “small renovation” of two Tulou buildings in Lantian Village, Longyan City. The project won the “New Into Old Excellent Renovation Award” recognized by The Architectural Review, a well-known British architectural magazine, in 2019. The success of the project has provided innovative practical experience for the renovation of other Tulou buildings. In this paper, we use this case to verify the possibility of applying extenics in Tulou building renovations, and the main purposes are as follows: (1) Extenics has strong analytical ability, and it can systematically analyze the overall structural elements of these two Tulou buildings, serving as a good tool for analyzing Tulou buildings. (2) Extenics transforms the thinking mode for solving contradictory problems found in the design of Tulou building renovations; thus, it provides the possibility of innovative Tulou building renovations, which is conducive to the sustainable renewal of Tulou buildings. (3) Through the demonstration of this case, it can be proven that extenics is fully applicable to solving contradictory problems between the protection and renovation of Tulou buildings.

**Figure 7:**
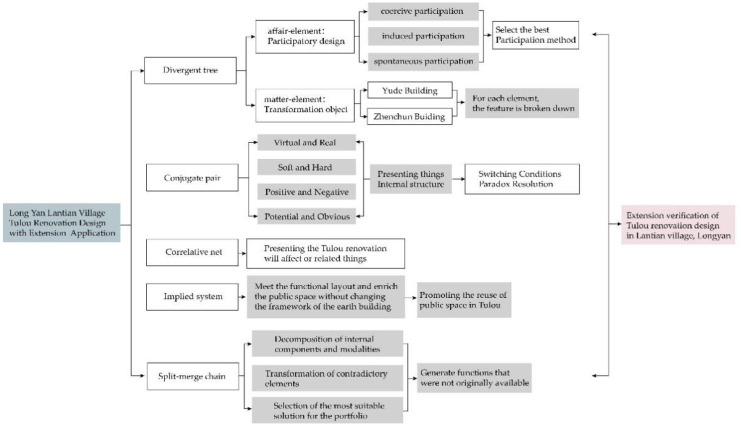
The technical framework.

Based on a basic element model, in the following sections, we systematically analyze and verify the feasibility and effectiveness of extenics through divergent tree, conjugate pair, correlative net, implied system, and split-merge chain analyses following the extension analysis method (the specific technical framework is shown in Figure 7); we discuss various possibilities of the expansion of the basic elements of Tulou buildings; and we stimulate people’s innovative thinking [43,44,45,46]. Hence, the results can guide sustainable updates of Tulou buildings that can adapt to social development.

### 3.1. Divergent Tree Analysis of Tulou Renovation Design

Generally speaking, things have many characteristics that can be expanded from the basic elements to a tree structure composed of multiple elements. A Tulou building is no exception. It is composed of multiple elements with the characteristics of multiple things and the values of multiple objects, which means that, starting from the conditions and objectives of Tulou buildings, the divergent tree structure composed of multiple elements can be expanded to formally describe the design of Tulou building renovations. Take the design of a Tulou renovation as an example, where the building, the Tulou, is a real thing, represented by the matter element (M); the participatory design is an event of subjective behavioral activity, represented by the affair element (A); and the relationship between the Tulou before and after renovation is represented by the relation element ®. First, the matter element (M) of the Tulou renovation is expanded, and its divergent tree can be expressed as:

(4) $$M=\left[\begin{array}{ccccc} O_{m} & , & C_{m1} & , & V_{m1}\\ & & C_{m2} & , & V_{m2}\\ & & C_{m3} & , & V_{m3}\\ & & C_{m4} & , & V_{m4}\\ & & C_{m5} & , & \left\{\begin{array}{c} V_{m51}\\ V_{m52} \end{array}\right.\\ & & C_{m6} & , & V_{m6}\\ & & C_{m7} & , & V_{m7}\\ & & C_{m8} & , & V_{m8}\\ & & C_{m9} & , & V_{m9}\\ & & C_{m10} & , & V_{m10}\\ & & \ldots \ldots & , & \ldots \ldots \end{array}\right]=\left[\begin{array}{ccccc} \mathrm{Reconstruction} & , & \mathrm{Dominantion}\;\mathrm{object} & , & \mathrm{Tulou}\\ & & \mathrm{Actuating}\;\mathrm{object} & , & \mathrm{Jun}\text{-}\mathrm{Han}\;\mathrm{Lin}’s\;\mathrm{Team}\\ & & \mathrm{Type} & , & \mathrm{Architectural}\;\mathrm{Heritage}\;\mathrm{Community}\\ & & \mathrm{Function} & , & \mathrm{Residence},\;\mathrm{defense}\\ & & \mathrm{Current}\;\mathrm{status} & , & \left\{\begin{array}{c} \mathrm{Deserted}\\ \mathrm{Idle} \end{array}\right.\\ & & \mathrm{Remodeling}\;\mathrm{method} & , & “\mathrm{Acupunctur}\;e\text{-}\mathrm{style}”\;\mathrm{therapy}\\ & & \mathrm{Design}\;\mathrm{method} & , & \mathrm{Participatory}\;\mathrm{design}\\ & & \mathrm{Participating}\;\mathrm{people} & , & \mathrm{Local}\;\mathrm{residents}\\ & & \mathrm{Location} & , & \mathrm{Longyan}\;\mathrm{Lantian}\;\mathrm{Village}\\ & & \mathrm{Time} & , & 2019\\ & & \ldots \ldots & , & \ldots \ldots \end{array}\right]=R$$

Various formal expressions of the first stage can be obtained from the divergence of R, shown as follows:

(5) $$M-|=\left[\begin{array}{ccccc} O_{m1} & , & C_{m11} & , & V_{m11}\\ & & C_{m12} & , & \left\{\begin{array}{c} V_{m121}\\ V_{m122} \end{array}\right.\\ & & C_{m13} & , & V_{m13}\\ & & C_{m14} & , & V_{m14}\\ & & C_{m15} & , & V_{m15}\\ & & C_{m16} & , & V_{m16}\\ & & \ldots \ldots & , & \ldots \ldots \end{array}\right]=\left[\begin{array}{ccccc} \mathrm{Reconstruction} & , & \mathrm{Dominant}\;\mathrm{object} & , & \mathrm{Yude}\;\mathrm{Building}\\ & & \mathrm{Actuating}\;\mathrm{object} & , & \left\{\begin{array}{c} \mathrm{Local}\;\mathrm{residents}\\ \mathrm{Jun}\text{-}\mathrm{Han}\;\mathrm{Lin}’s\;\mathrm{Team} \end{array}\right.\\ & & \mathrm{Current}\;\mathrm{status} & , & \mathrm{Has}\;\mathrm{been}\;\mathrm{converted}\;\mathrm{into}\;a\;\mathrm{school}\\ & & \mathrm{Functions}\;\mathrm{before}\;\mathrm{remodeling} & , & \mathrm{Residence}\\ & & \mathrm{Remodeled}\;\mathrm{functions} & , & \mathrm{Summer}\;\mathrm{Camp}\\ & & \mathrm{Remodeling}\;\mathrm{method} & , & \mathrm{Spatial}\;\mathrm{reorganization}\\ & & \ldots \ldots & , & \ldots \ldots \end{array}\right]=R_{1}$$

(6) $$M-|=\left[\begin{array}{ccccc} O_{m2} & , & C_{m21} & , & V_{m21}\\ & & C_{m22} & , & V_{m22}\\ & & C_{m23} & , & V_{m23}\\ & & C_{m24} & , & \left\{\begin{array}{c} V_{m241}\\ V_{m242} \end{array}\right.\\ & & C_{m25} & , & V_{m25}\\ & & C_{m26} & , & V_{m26}\\ & & \ldots \ldots & , & \ldots \ldots \end{array}\right]=\left[\begin{array}{ccccc} \mathrm{reconstruction} & & \mathrm{Dominant}\;\mathrm{object} & , & \mathrm{Zhenchun}\;\mathrm{building}\\ & & \mathrm{Actuating}\;\mathrm{object} & , & \mathrm{Jun}\text{-}\mathrm{Han}\;\mathrm{Lin}’s\;\mathrm{Team}\\ & & \mathrm{Current}\;\mathrm{status} & , & \mathrm{deserted}\\ & & \mathrm{Reason} & , & \left\{\begin{array}{c} \mathrm{Equipment}\;\mathrm{lag}\\ \mathrm{Large}\;\mathrm{number}\;\mathrm{of}\;\mathrm{residents}\;\mathrm{moved}\;\mathrm{out} \end{array}\right.\\ & & \mathrm{Design}\;\mathrm{method} & , & \mathrm{Participatory}\;\mathrm{Design}\\ & & \mathrm{Participants} & , & \mathrm{Local}\;\mathrm{residents}\\ & & \ldots \ldots & , & \ldots \ldots \end{array}\right]=R_{2}$$

The divergent expression of the second stage can also be obtained from R_1_, shown as follows:

(7) $$M-|=\left[\begin{array}{ccccc} O_{m1} & , & C_{m111} & , & V_{m111}\\ & & C_{m112} & , & \left\{\begin{array}{c} V_{m1121}\\ V_{m1122}\\ V_{m1123}\\ V_{m1124} \end{array}\right.\\ & & C_{m113} & , & \left\{\begin{array}{c} V_{m1131}\\ V_{m1132} \end{array}\right.\\ & & C_{m114} & , & \left\{\begin{array}{c} V_{m1141}\\ V_{m1142}\\ V_{m1143} \end{array}\right.\\ & & \ldots \ldots & , & \ldots \ldots \end{array}\right]=\left[\begin{array}{ccccc} \mathrm{reconstruction} & , & \mathrm{Dominant}\;\mathrm{object} & , & \mathrm{Architectural}\;\mathrm{interiors}\\ & & \mathrm{Control}\;\mathrm{objects} & , & \left\{\begin{array}{c} \mathrm{Living}\;\mathrm{room}\\ \mathrm{Kitchen}\\ \mathrm{Storage}\;\mathrm{room}\\ \mathrm{Bedroom} \end{array}\right.\\ & & \mathrm{Methods} & , & \left\{\begin{array}{c} \mathrm{New}\;\mathrm{function}\;\mathrm{coupling}\\ \mathrm{Spatial}\;\mathrm{Reorganization} \end{array}\right.\\ & & \mathrm{Remodeled}\;\mathrm{functions} & , & \left\{\begin{array}{c} \mathrm{Classroom}\\ \mathrm{Student}\;\mathrm{Dormitory}\\ \mathrm{Public}\;\mathrm{Library} \end{array}\right.\\ & & \ldots \ldots & , & \ldots \ldots \end{array}\right]=R_{11}$$

(8) $$M-|=\left[\begin{array}{ccccc} O_{m1} & , & C_{m121} & , & V_{m121}\\ & & C_{m122} & , & \left\{\begin{array}{c} V_{m1221}\\ V_{m1222} \end{array}\right.\\ & & C_{m123} & , & \left\{\begin{array}{c} V_{m1231}\\ V_{m1232} \end{array}\right.\\ & & C_{m124} & , & \left\{\begin{array}{c} V_{m1241},V_{m1241}\\ V_{m1242},V_{m1242} \end{array}\right.\\ & & \ldots \ldots & , & \ldots \ldots \end{array}\right]=\left[\begin{array}{ccccc} \mathrm{reconstruction} & , & \mathrm{Dominant}\;\mathrm{object} & , & \mathrm{Building}\;\mathrm{exterior}\\ & & \mathrm{Control}\;\mathrm{objects} & , & \left\{\begin{array}{c} \mathrm{Front}\;\mathrm{yard}\\ \mathrm{Tulou}\;\mathrm{exterior}\;\mathrm{wall} \end{array}\right.\\ & & \mathrm{Methods} & , & \left\{\begin{array}{c} \mathrm{Adding}\;\mathrm{funnel}\text{-}\mathrm{shaped}\;\mathrm{steps}\\ \mathrm{Adding}\;a\;\mathrm{room} \end{array}\right.\\ & & \mathrm{Remodeled}\;\mathrm{functions} & , & \left\{\begin{array}{c} \mathrm{Reading}\;\mathrm{room},\;\mathrm{Audience}\;\mathrm{seats}\\ \mathrm{Washroom},\;\mathrm{Shower}\;\mathrm{room} \end{array}\right.\\ & & \ldots \ldots & , & \ldots \ldots \end{array}\right]=R_{12}$$

If the internal part of the Zhenchun Building is renovated into a B&B hotel or folk museum, R_2_ can be further divergent, and its expression is as follows:

(9) $$M-|=\left[\begin{array}{ccccc} O_{m2} & , & C_{m211} & , & V_{m211}\\ & & C_{m212} & , & \left\{\begin{array}{c} V_{m2121}\\ V_{m2122}\\ V_{m2123}\\ V_{m2124} \end{array}\right.\\ & & C_{m213} & , & \left\{\begin{array}{c} V_{m2131}\\ V_{m2132} \end{array}\right.\\ & & C_{m214} & , & \left\{\begin{array}{c} V_{m2141}\\ V_{m2142} \end{array}\right.\\ & & \ldots \ldots & , & \ldots \ldots \end{array}\right]=\left[\begin{array}{ccccc} \mathrm{reconstruction} & , & \mathrm{Dominant}\;\mathrm{object} & , & \mathrm{Architectural}\;\mathrm{interiors}\\ & & \mathrm{Control}\;\mathrm{objects} & , & \left\{\begin{array}{c} \mathrm{Living}\;\mathrm{room}\\ \mathrm{Kitchen}\\ \mathrm{Storage}\;\mathrm{room}\\ \mathrm{Bedroom} \end{array}\right.\\ & & \mathrm{Methods} & , & \left\{\begin{array}{c} \mathrm{New}\;\mathrm{function}\;\mathrm{coupling}\\ \mathrm{Spatial}\;\mathrm{Reorganization} \end{array}\right.\\ & & \mathrm{Remodeled}\;\mathrm{functions} & , & \left\{\begin{array}{c} B\text{\&}B\;\mathrm{Hotels}\\ \mathrm{Folklore}\;\mathrm{Museum} \end{array}\right.\\ & & \ldots \ldots & , & \ldots \ldots \end{array}\right]=R_{21}$$

(10) $$M-|=\left[\begin{array}{ccccc} O_{m2} & , & C_{m221} & , & V_{m221}\\ & & C_{m222} & , & V_{m222}\\ & & C_{m223} & , & V_{m223}\\ & & C_{m224} & , & V_{m224}\\ & & \ldots \ldots & , & \ldots \ldots \end{array}\right]=\left[\begin{array}{ccccc} \mathrm{reconstruction} & , & \mathrm{Dominant}\;\mathrm{object} & , & \mathrm{Building}\;\mathrm{exterior}\\ & & \mathrm{Control}\;\mathrm{objects} & , & \mathrm{Atrium}\\ & & \mathrm{Methods} & , & \mathrm{Adding}\;a\;\mathrm{spiral}\;\mathrm{staircase}\;\text{-}\;“\mathrm{High}\;\mathrm{Tower}”\\ & & \mathrm{Remodeled}\;\mathrm{functions} & , & \mathrm{Observation}\;\mathrm{Tower}\\ & & \ldots \ldots & , & \ldots \ldots \end{array}\right]=R_{22}$$

**Figure 8:**
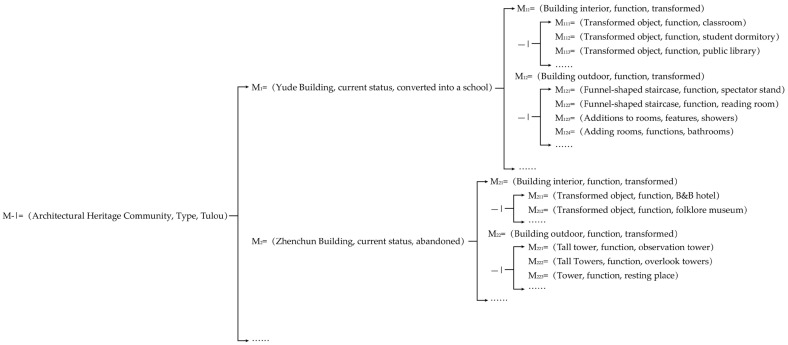
The divergent tree of the Tulou building renovation case.

From the above analysis of the design of the Tulou building renovation, the divergent tree can be summarized as shown in Figure 8:

In addition, the microregeneration of Tulou buildings assisted by the participatory design in this case can be analyzed by the affair element (A), which can be expressed as:

(11) $$A=\left[\begin{array}{ccccc} O_{a} & , & C_{a1} & , & V_{a1}\\ & & C_{a2} & , & V_{a2}\\ & & C_{a3} & , & V_{a3}\\ & & \ldots \ldots & , & \ldots \ldots \end{array}\right]=\left[\begin{array}{ccccc} \mathrm{Participatory}\;\mathrm{design} & , & \mathrm{Dominant}\;\mathrm{object} & , & \mathrm{Design}\;\mathrm{scheme}\\ & & \mathrm{Actuating}\;\mathrm{object} & , & \mathrm{The}\;\mathrm{People}\\ & & \mathrm{Types} & , & 3\\ & & \ldots \ldots & , & \ldots \ldots \end{array}\right]=N$$

(12) $$A-|=\left[\begin{array}{ccccc} O_{a} & , & C_{a11} & , & V_{a11}\\ & & C_{a12} & , & V_{a12}\\ & & C_{a13} & , & V_{a13}\\ & & C_{a14} & , & V_{a14}\\ & & C_{a15} & , & V_{a15}\\ & & C_{a16} & , & V_{a16}\\ & & \ldots \ldots & , & \ldots \ldots \end{array}\right]=\left[\begin{array}{ccccc} \mathrm{Participatory}\;\mathrm{design} & , & \mathrm{Forms} & , & \mathrm{Coercive}\;\mathrm{participation}\\ & & \mathrm{Dominant}\;\mathrm{object} & , & \mathrm{Local}\;\mathrm{residents}\\ & & \mathrm{Features} & , & \mathrm{Briefly}\;\mathrm{inform}\;\mathrm{the}\;\mathrm{renovation}\;\mathrm{plan}\\ & & \mathrm{Existing}\;\mathrm{problems} & , & \mathrm{Lack}\;\mathrm{of}\;\mathrm{autonomy}\\ & & \mathrm{Participation}\;\mathrm{level} & , & \mathrm{Low}\;\mathrm{level}\\ & & \mathrm{Effects} & , & \mathrm{Poorly}\\ & & \ldots \ldots & , & \ldots \ldots \end{array}\right]=N_{1}$$

(13) $$A-|=\left[\begin{array}{ccccc} O_{a} & , & C_{a21} & , & V_{a21}\\ & & C_{a22} & , & \left\{\begin{array}{c} V_{a221}\\ V_{a222} \end{array}\right.\\ & & C_{a23} & , & V_{a23}\\ & & C_{a24} & , & V_{a24}\\ & & C_{a25} & , & V_{a25}\\ & & C_{a26} & , & V_{a26}\\ & & \ldots \ldots & , & \ldots \ldots \end{array}\right]=\left[\begin{array}{ccccc} \mathrm{Participatory}\;\mathrm{design} & , & \mathrm{Forms} & , & \mathrm{Induced}\;\mathrm{participation}\\ & & \mathrm{Dominant}\;\mathrm{object} & , & \left\{\begin{array}{c} \mathrm{Local}\;\mathrm{residents}\\ \mathrm{Community}\;\mathrm{Managers} \end{array}\right.\\ & & \mathrm{Features} & , & \mathrm{Have}\;a\;\mathrm{voice}\\ & & \mathrm{Existing}\;\mathrm{problems} & , & \mathrm{No}\;\mathrm{real}\;\mathrm{power}\;\mathrm{in}\;\mathrm{the}\;\mathrm{decision}\text{-}\mathrm{making}\;\mathrm{process}\\ & & \mathrm{Participation}\;\mathrm{level} & , & \mathrm{Intermediate}\\ & & \mathrm{Effects} & , & \mathrm{General}\;\mathrm{effect}\\ & & \ldots \ldots & , & \ldots \ldots \end{array}\right]=N_{2}$$

(14) $$A-|=\left[\begin{array}{ccccc} O_{a} & , & C_{a31} & , & V_{a31}\\ & & C_{a32} & , & \left\{\begin{array}{c} V_{a321}\\ V_{a322}\\ V_{a323} \end{array}\right.\\ & & C_{a33} & , & \left\{\begin{array}{c} V_{a331}\\ V_{a332}\\ V_{a333} \end{array}\right.\\ & & C_{a34} & , & V_{a34}\\ & & C_{a35} & , & V_{a35}\\ & & C_{a36} & , & V_{a36}\\ & & \ldots \ldots & , & \ldots \ldots \end{array}\right]=\left[\begin{array}{ccccc} \mathrm{Participatory}\;\mathrm{design} & , & \mathrm{Forms} & , & \mathrm{Spontaneous}\;\mathrm{participation}\\ & & \mathrm{Dominant}\;\mathrm{object} & , & \left\{\begin{array}{c} \mathrm{Local}\;\mathrm{residents}\\ \mathrm{Community}\;\mathrm{managers}\\ \mathrm{Related}\;\mathrm{professionals} \end{array}\right.\\ & & \mathrm{Features} & , & \left\{\begin{array}{c} \mathrm{Shared}\;\mathrm{decision}\;\mathrm{making}\\ \mathrm{Co}\text{-}\mathrm{design}\\ \mathrm{Co}\text{-}\mathrm{Management} \end{array}\right.\\ & & \mathrm{Existing}\;\mathrm{problems} & , & \mathrm{Need}\;\mathrm{to}\;\mathrm{explain}\;\mathrm{the}\;\mathrm{relevant}\;\mathrm{knowledge}\;\mathrm{to}\;\mathrm{residents}\\ & & \mathrm{Participation}\;\mathrm{Level} & , & \mathrm{Senior}\\ & & \mathrm{Effects} & , & \mathrm{Very}\;\mathrm{good}\\ & & \ldots \ldots & , & \ldots \ldots \end{array}\right]=N_{3}$$

**Figure 9:**
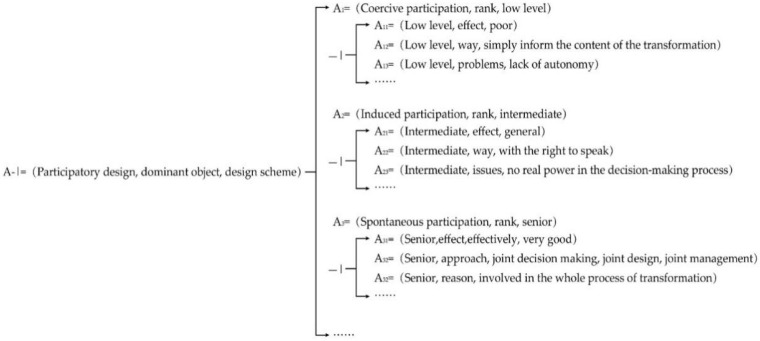
The divergent tree of the participatory design.

The divergent tree of the event of the participatory design in this case can be shown as Figure 9:

The relation element of the renovation project can be expressed as:

(15) $$R=\left[\begin{array}{ccccc} O_{r} & , & C_{r1} & , & \left\{\begin{array}{c} V_{r11}\\ V_{r12} \end{array}\right.\\ & & C_{r2} & , & V_{r2}\\ & & C_{r3} & , & V_{r3}\\ & & \ldots \ldots & , & \ldots \ldots \end{array}\right]=\left[\begin{array}{ccccc} \mathrm{Transformation}\;\mathrm{of}\;\mathrm{relationships} & , & \mathrm{Before}\;\mathrm{remodeling} & , & \left\{\begin{array}{c} \mathrm{Deserted}\\ \mathrm{Idle} \end{array}\right.\\ & & \mathrm{After}\;\mathrm{remodeling} & , & \mathrm{Space}\;\mathrm{Reuse}\\ & & \mathrm{Effects} & , & \mathrm{Promoting}\;\mathrm{sustainable}\;\mathrm{development}\;\mathrm{of}\;\mathrm{Tulou}\\ & & \ldots \ldots & , & \ldots \ldots \end{array}\right]$$

From the above divergent tree analysis, it can be seen that when renovating Tulou buildings, functional types of the buildings can be expanded at multiple levels for the consideration of various possibilities. From the affair element analysis of the participatory design, it can be seen that spontaneous participation of local communities in the renovation obtains the best effect. Therefore, in a sustainable renovation plan of architectural heritage sites, local communities play an important role in the revitalization and maintenance of architectural heritage sites; thus, the participation of local communities is crucial [47]. This spontaneous participation in the design process can enhance residents’ abilities to improve the quality of community life and art, and can stimulate villagers to see the infinite possibilities of Tulou building renovations. Therefore, the divergent tree method can be applied to expand the analysis and vertical thinking for the design of Tulou renovations, to obtain a specific and detailed understanding of the Tulou as a renovation object. The maximum space of the Tulou can be developed and its transformable prospects can be fully excavated, further laying a solid foundation for effectively driving innovation generation.

### 3.2. Conjugate Pair Analysis of Tulou Renovation Design

In extenics, conjugation exists in pairs, which are called conjugate pairs. Everything (O_m_) has four pairs of attributes, namely virtual part (im) and real part (re), negative part (ngc) and positive part (psc), potential part (it) and obvious part (ap), as well as soft part (sf) and hard part (hr), expressed as O_m_ = re (O_m_) +im (O_m_) = ngc (O_m_) +psc (O_m_) = it (O_m_) +ap (O_m_) = sf (O_m_) +hr (O_m_). They can be converted to each other under certain conditions. Through the analysis of conjugate pairs, the composition of things can be described more comprehensively, systematically, and accurately. Various problems will be encountered in the renovation of Tulou buildings. For example, Tulou buildings cannot meet the needs of modern life, leading users to refuse to use Tulou buildings. Therefore, the renovation problem that should be considered is: How can we renovate and reorganize the spatial layout of Tulou buildings to meet the modern life needs of people? Through investigation, the basic conditions of the Yude Building and the Zhenchun Building can be identified. The favorable condition is that the building frames of the two Tulou buildings are still intact, whereas the unfavorable condition is that the equipment in the Tulou buildings lag behind, and some basic functions cannot be realized, such as toilets and shower rooms that do not meet the living requirements of local residents. Hence, in the design of the renovations, the focus is to revitalize the Tulou buildings and to realize sustainable renewal. In the thinking process, the contradictory problem can be solved by using a conjugate pair analysis.

Through an in-depth analysis of the conjugate pairs of the Tulous buildings and the transformation of the conjugate part, the contradictory problems can be solved to achieve the purpose of innovation. There are various limitations in renovation work under the historical background of Tulou buildings. Therefore, a comprehensive and overall analysis of the Tulou buildings must be conducted, with regards to the process of protection, renovation, and renewal as a whole design process with conjugate thinking [6]. The transformation analysis of conjugate pairs makes it possible to transform the unfavorable conditions of idle space in Tulou buildings into the favorable conditions of active space required for renewal. According to the principle of conjugate pair analysis, in this paper, we take the renovations of the Yude Building and the Zhenchun Building as the examples to analyze their conjugate pairs. Among them, such material parts as the architectural heritage entities of the Tulou buildings and the physical objects preserved by culture belong to the real part of the virtual-real conjugate pair, while such immaterial parts as the spatial scale, function, and image of the architectural space belong to the virtual part. Folk culture, spatial function, and other human behavioral activities, or the relationship with the hard part belong to the soft part of the soft and hard conjugate pair, and the part of the Tulou building entity and the village environment that make up the overall building of the Tulou belong to the hard part. The unexplored history and culture or the hints and feelings brought belong to the potential part of the potential and obvious conjugate pair, while the exterior of the building and the developed building, which can be intuitively or obviously perceived, belong to the obvious part.The economic benefits or favorable conditions brought by tourism and other resources belong to the positive part of the positive and negative conjugate pair, while the costs paid for the restoration of the tulou buildings or the unfavorable conditions such as the destruction of buildings and the loss of talents belong to the negative part. The specific conjugation pairs are shown in Table 2.

For example, the soft and hard conjugate pair of a Tulou building can be expressed as:

(16) $$\begin{array}{l} M=M_{sf}+M_{hr}=\left[\begin{array}{ccccc} sf(O_{m}) & , & C_{m1_{i}{}_{m}} & , & V_{m1_{i}{}_{m}}\\ & & C_{m2_{i}{}_{m}} & , & V_{m2_{i}{}_{m}}\\ & & C_{m3_{i}{}_{m}} & , & V_{m3_{i}{}_{m}} \end{array}\right]+\left[\begin{array}{ccccc} hr(O_{m}) & , & C_{m1_{hr}} & , & V_{m1_{hr}}\\ & & C_{m2_{hr}} & , & V_{m2_{hr}} \end{array}\right]\\ =\left[\begin{array}{ccccc} \mathrm{Soft} & , & \mathrm{Types} & , & \mathrm{History}\;\mathrm{and}\;\mathrm{Culture}\\ & & \mathrm{Types} & , & \mathrm{Folk}\;\mathrm{culture}\\ & & \mathrm{Types} & , & \mathrm{Hakka}\;\mathrm{culture} \end{array}\right]+\left[\begin{array}{ccccc} \mathrm{Hard} & , & \mathrm{Types} & , & \mathrm{Tulou}\;\mathrm{Architectural}\;\mathrm{Entity}\\ & & \mathrm{Types} & , & \mathrm{Village}\;\mathrm{Environment} \end{array}\right] \end{array}$$

According to the analysis of the soft and hard parts of the Tulou buildings, taking the toilets and shower rooms added by the local residents in the Yude Building as an example, the added rooms are the hard part, and the color relationship between the added buildings and the outer wall of the Tulou building is the soft part; thus the soft and hard conjugate pair transformation analysis can be carried out. First, the soft and hard conjugate pair analysis is conducted:

(17) $$\begin{array}{l} M_{hr1}=\left[\begin{array}{ccccc} \mathrm{Exterior}\;\mathrm{wall}\;\mathrm{color}\;\mathrm{of}\;\mathrm{the}\;\mathrm{extension}\;\mathrm{house} & , & \mathrm{Types} & , & \mathrm{Bathroom}\;\mathrm{exterior}\;\mathrm{wall}\;\mathrm{color}\\ & & \mathrm{Types} & , & \mathrm{Shower}\;\mathrm{exterior}\;\mathrm{wall}\;\mathrm{color} \end{array}\right]\\ M_{hr2}=\left[\begin{array}{ccccc} \mathrm{Tulou}\;\mathrm{exterior}\;\mathrm{wall} & , & \mathrm{Types} & , & \mathrm{Exterior}\;\mathrm{wall}\;\mathrm{color} \end{array}\right]\\ M_{sf}=\left[\begin{array}{ccccc} \mathrm{Conflict}\;\mathrm{Relationships} & , & M_{hr1} & , & \mathrm{Exterior}\;\mathrm{wall}\;\mathrm{color}\;\mathrm{of}\;\mathrm{the}\;\mathrm{extension}\;\mathrm{house}\\ & & M_{hr2} & , & \mathrm{Tulou}\;\mathrm{exterior}\;\mathrm{wall}\;\mathrm{color} \end{array}\right] \end{array}$$

Then, the soft and hard conjugate transformation is conducted:

(1) Transformation is conducted to the hard part M_hr1_ and it changed into M_hr_’
  (18) $$M_{hr}’=\left[\begin{array}{ccccc} \mathrm{Exterior}\;\mathrm{wall}\;\mathrm{color}\;\mathrm{of}\;\mathrm{the}\;\mathrm{extension}\;\mathrm{house} & , & \mathrm{Processing} & , & \mathrm{Painted}\;\mathrm{with}\;a\;\mathrm{color}\;\mathrm{similar}\;\mathrm{to}\;\mathrm{rammed}\;\mathrm{earth} \end{array}\right]$$
(2) The transformation of the hard part causes the transformation of the soft part Msf’’
  (19) $$M_{sf}”=\left[\begin{array}{ccccc} \mathrm{Fusion}\;\mathrm{relationship} & , & M’ & , & \mathrm{Exterior}\;\mathrm{wall}\;\mathrm{color}\;\mathrm{of}\;\mathrm{the}\;\mathrm{extension}\;\mathrm{house}\\ & & M_{hr2} & , & \mathrm{Tulou}\;\mathrm{exterior}\;\mathrm{wall}\;\mathrm{color} \end{array}\right]$$

From the perspective of the above-mentioned soft and hard conjugation, if the hard part of the elevation color of the additional building changes, the relevant soft part will also change accordingly, which skillfully resolves the conflict between the elevation color of the local residents’ own additional building changes and the exterior wall color of the Tulou building, as well as to integrate the additional building changes and the exterior wall of the Tulou building by adopting transformation thinking. In addition to the analysis from the perspective of soft and hard conjugation, it can also be analyzed from other perspectives. Therefore, applying conjugate pair thinking can enable the relevant designers to understand the internal structure of a Tulou building more comprehensively, to analyze its strengths and weaknesses comprehensively, and to solve the contradictory problems in the design process of the Tulou building renovations in a targeted manner according to the inter-convertibility of the conjugate parts under certain conditions, and therefore, to provide sufficient prerequisites when the Tulou building is renovated.

### 3.3. Correlative Net Analysis of Tulou Renovation Design

**Figure 10:**
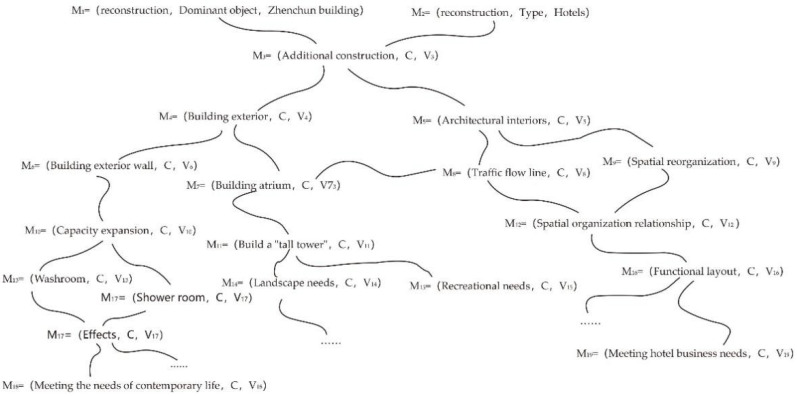
The correlative net analysis of the Zhenchun Building.

In this case, it can be seen that the Yude Building was transformed into a school before the renovations, then we analyzed the Zhenchun Building. If we intend to transform the Zhenchun Building into a hotel or B&B, the design requirement is to meet the functional layout needs of modern hotels as much as possible without changing the architectural framework of the Tulou building. Since the building floors and building frames of the Tulou building are fixed, more factors such asthe spatial layout must be considered in the renovation to enrich the design. The correlative net is shown in Figure 10. We can consider how to meet the basic needs from the elements in the correlative net. Therefore, by applying a correlative net analysis in the design of Tulou building renovations we can fully consider the correlation between the Tulou ontology and the target demand, so that this renovation target can better match with the ontology and can provide the main idea for determining the purpose of the Tulou renovation.

### 3.4. Implied System Analysis of Tulou Renovation Design

**Figure 11:**
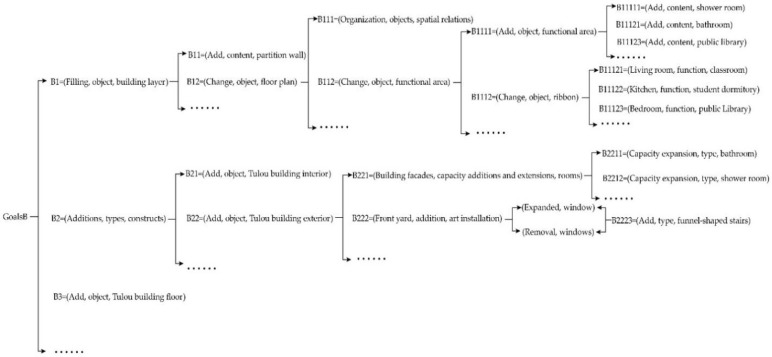
The implied system analysis of the Yude Building.

Taking the renovation of the Tulou buildings as an example, goal B refers to the renovation and repair of the Tulou buildings without changing their original structures and living environments, to enrich the public activity space and to promote the reuse and sustainable renewal of the Tulou buildings. By conducting an implied system analysis, we can consider and analyze problems at the next level to solve problems at the previous level. Since Tulou buildings are precious architectural heritage sites, more attention should be paid to the authenticity and historical value of these architectural heritage sites when repairing, which results in strict restrictions on the renovations of the Tulou buildings. In order to achieve this goal, Lin Junhan’s team proposed that the walls, floors, stairs, corners, roofs, entrances, and windows of the Tulou buildings could be renovated, and the goal of the next level could be achieved by adding structures and coupling new functions. However, not all the next-level goals could be achieved, and the final goal of the lowest level could only be achieved by screening. The renovated Yude Building is used for the reverse-thinking reasoning analysis, and the renovation process is represented by using a formal implied system analysis. The specific and implied system analysis is shown in Figure 11. Conducting an implied system analysis of the renovation design for a Tulou building is the key to determining the renovation plan of the Tulou building by taking the renovation goal as the upper-level goal, analyzing the next level goal around the upper-level goal under the overall control, and filtering out the final goal that can be achieved.

### 3.5. Split-Merge Chain Analysis of Tulou Renovation Design

Taking the renovation of the Yude Building as an example, the main functions of the Yude Building after renovation included a summer camp site, student dormitory, public library, audience seats, bathrooms, shower rooms, etc. According to their own needs, local residents changed the functions of the Yude Building from an original living space to a school for holding summer camps. They renovated the original bedrooms, kitchens, living rooms, storage rooms, etc. of the Tulou building, and decomposed and reorganized the building space to make the functions of the building satisfy their needs. Afterwards, according to the needs of the local residents, once agina, the Lin Junhan’s team implanted creative “art installations” in the front yard of the Tulou, and transformed the rooms connecting with the front yard into a public library, enriching the public space as well as activating the heritage community and interactions among people. For example, in the renovation of the Yude Building, according to the principle of a split-merge chain analysis and certain conditions, it can be decomposed into several elements; the model can be expressed as: M = M//{M_1_, M_2_, M_3_, M_4_, M_5_, M_6_, M_7_} = (Yude Building, function, summer camp classroom, student dormitory, public library, audience seats, reading room, bathroom, shower room))//{(living room on the first floor, function, summer camp classroom), (kitchen on the first floor, function, student dormitory), (bedroom on the second floor, function, public library), (funnel staircase, function, audience seats), (funnel staircase, function, reading room), (additional room, function, bathroom), (additional room, function, shower room)}. For the Tulou building renovation, by using the split-merge chain analysis, it is possible to combine the Tulou with other things to form new things, and also to decompose the whole Tulou into several different new things, so that the Tulou building has some characteristics that it did not have originally, solving the existing contradictory problems of the Tulou building and assisting the renovation of the Tulou building with new ideas and in line with the actual needs.

In summary, through the above divergent tree, conjugate pair, correlative net, implied system and split-merge chain analyses of the Lin Junhan team’s Tulou renovation projects, we verified that extenics theory can play an important role in Tulou building renovations, and we also confirmed its effectiveness and feasibility.

## 4. Conclusions and Implication

### 4.1. Exploring the Regeneration Path of a Tulou from an Extenics Perspective

With the influence of multiple aspects such as people’s lifestyles and social development, many traditional dwellings such as Tulou buildings are not able to continue to meet the housing needs of modern people due to some problems. A large number of residents move out of Tulou buildings, resulting in the serious phenomenon of Tulou “emptiness”, which has resulted in Tulou buildings gradually declining due to the lack of daily maintenance by users. In addition, over time, the walls, surfaces, and other infrastructures of Tulou buildings have been damaged, to varying degrees, by human behaviors, wind and rain erosion, earthquakes, and other natural disasters, and the number of buildings has decreased significantly, resulting in the gradual disappearance of the Tulou buildings’ cultural history. With the rapid development of Tulou tourism, the reuse of Tulou space has usually been based on commercial development, display, and other functional transformations and less consideration has been given to Tulou buildings in non-popular scenic spots; thus, a large number of ordinary Tulou buildings have been idle and abandoned, and space resources have been wasted. Therefore, in-depth thinking and further research is needed to better protect and activate Tulou buildings, to explore their regeneration paths, to improve their living environment, and to meet the living needs of modern people, and therefore to achieve the desired sustainability [48].

For a long time, only natural language could accurately describe the protection of architectural heritage. However, natural language has significant limitations, such as sensibility, non-standardization, randomness, and fuzziness, which makes it difficult to express many problems accurately. It has been found that extenics, which is used to solve complex contradictory problems, can improve the design of Tulou building renovations, interpret the renovation process in a rigorous structural system, and can express it in a formal way. It can promote the white-box creative process and can provide reliable basic tools for Tulou building renovation, making the renovation process more intuitive and rigorous and promoting the scientific renovation of Tulou buildings. Through the extension verification of the renovation of Tulou buildings in the previous sections, the feasibility of extenics in Tulou architecture is confirmed. Therefore, this paper begins to explore the regeneration path of Tulou from the perspective of extenics.

#### 4.1.1. Renovation Design System of Tulou Buildings from the Perspective of Extenics

**Figure 12:**
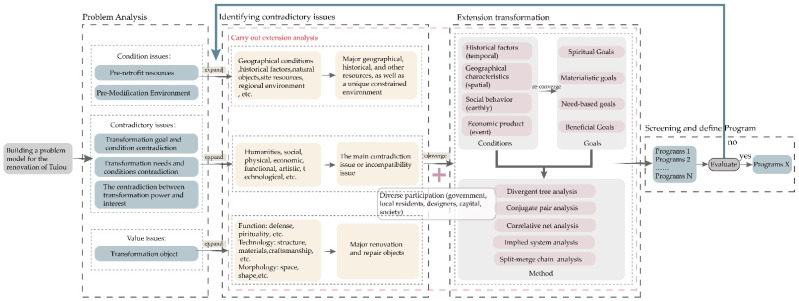
The renovation design system of the Tulou buildings [17].

**Figure 13:**
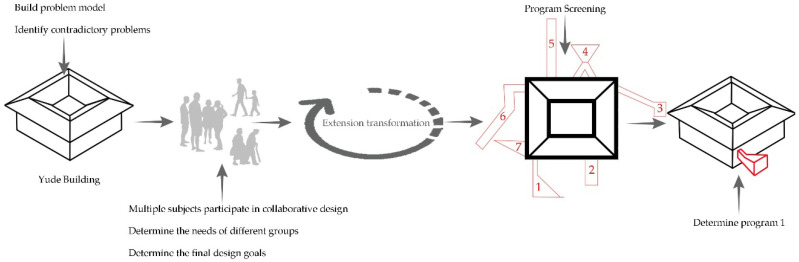
Demonstration process of the space renovation in the Yude Building.

Under different historical backgrounds, extenics provides unique modes of thinking for the renovation and innovation of Tulou buildings to solve problems resulting from contradictions and different conditions. The innovative procedures in the extension analysis method should be fully applied to implement the concept of non-value-added design into the renovation and renewal of Tulou buildings, and to build a replicable design system for Tulou renovations (Figure 12). As shown in the figure, the first step should be to identify and define the problems to build a problem model for the design of Tulou building renovations and to identify the contradictions. The second step is to carry out a problem analysis, to confirm the conditions and objectives of complex contradictions in the renovation of Tulou buildings, and to conduct an extension analysis method and conjugate pair analysis. The third step is to analyze the different needs and conditions of various subjects through the participation of multiple subjects to determine the final goal, and to carry out extension transformation on the main contradictory problems. Finally, different renovation plans are obtained through extension transformation. After selection, the final plan can be determined if it is the most suitable one. If not, the extension analysis method should be conducted again after returning to the second step. This procedure shows that it is feasible to creatively apply extenics in customizing designs for the protection and renovation of Tulou buildings. Meanwhile, the renovation design system is also applicable to the renovation and repair of other architectural heritage sites in addition to Tulou buildings. The example of the Yude Building renovations was used to demonstrate and analyze the relevance of the renovation design system to the spaces involved, as shown in Figure 13.

#### 4.1.2. Suggestions on the Regeneration Path of Tulou Buildings

##### (1) Multiple Participation as well as Reasonable Allocation and Reorganization of Human and Material Resources

**Figure 14:**
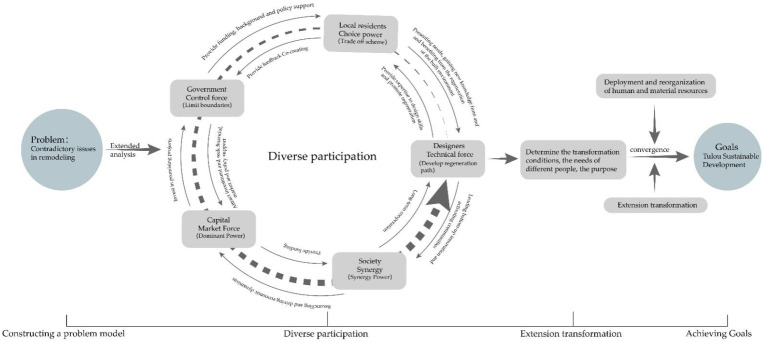
Extension analysis diagram of multiple participation [50].

The sustainable preservation and development of Tulou buildings can only be realized on the basis of renovation, activation and protection with the forces of multiple parties, combined with the theory of extenics (as shown in Figure 14) and giving full play to the role of multiple subjects [49]. First of all, problems should be identified to establish an extension problem model and to identify the major contradictory problems, and an extension analysis should be carried out. Secondly, the positions and needs of local residents, governments, designers and other different subjects should be fully understood to build a joint force for the regeneration of Tulou buildings and their built environment, achieving the purpose of multiple participation in the renovation. Finally, according to the needs of multiple subjects, the extension method can be used to carry out extension transformation, promoting reasonable allocation and reorganization of human and material resources in the Tulou building renovations, as well as to completing the ultimate goal. The projects of renovation and renewal of Tulou buildings require the guidance of experts and professionals with relevant professional knowledge, reasonable control and policy support of the government, and funds for the renovation and repair of Tulou buildings provided by the capital market. Reasonable allocation and reorganization of human and material resources, and the coordination of social forces that arouse the villagers’ awareness of protection are essential to establish an effective concept of cultural protection, thus promoting the sustainable development of Tulou buildings.

##### (2) Solving the power and interest contradiction between local residents and relevant government departments

**Figure 15:**
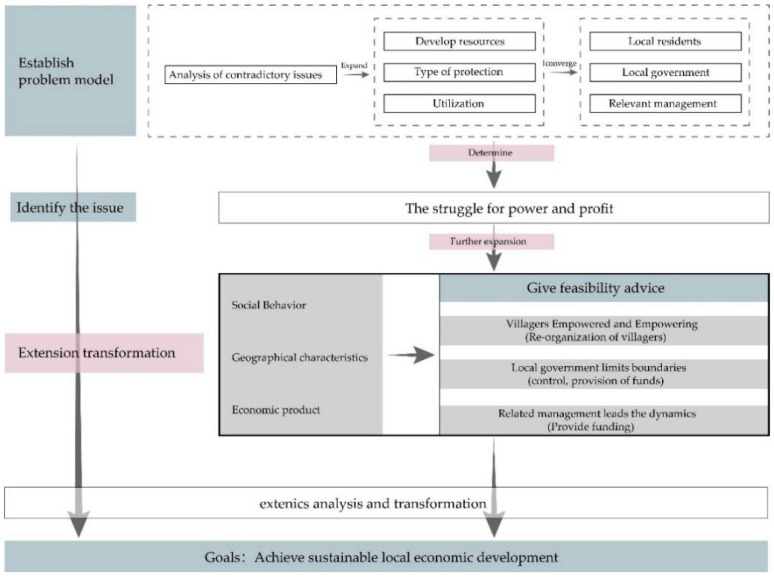
Path to solve power contradictions.

From the perspectives of historical and cultural inheritance and development, the carrier of Tulou culture is the local residents. However, from the perspectives of development, protection, and utilization, the main subjects of specific behavior are local government and relevant management. Therefore, we have good reasons to explain that competing for Tulou cultural resources and interests is not the competition for the orthodox status of folk culture inheritance and its legalized position, but mainly the power and interest contradictions between the local government and relevant management departments and the local residents [51]. The major reasons are the imperfections of the relevant laws, the lack of long-term strategic development vision, and the blind adoption of protective measures. Therefore, a model of power and interest contradictions between local residents and relevant governmental departments can be built according to extenics theory (Figure 15), after which the main contradictory problems can be determined, and the extension conditions and objectives can be transformed. Moreover, villagers can be empowered and reorganized to abide by the boundaries limited by the local government and the leadership of relevant management departments; thus, the goal aimed at sustainable development of local economy can be finally achieved. The power and interest contradictions between local residents and relevant government departments must be solved. If this is not handled well, it will be detrimental to the reasonable development and reuse of historical and cultural resources, affecting the sustainable development of the local social economy.

##### (3) Microspace Re-creation

**Figure 16:**
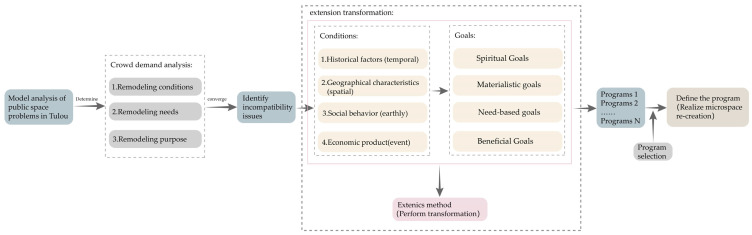
Micro-space re-creation from the perspective of extenics.

Through the design thinking of extenics, an extension analysis can be carried out of the shared spaces of Tulou buildings to achieve the purpose of space regeneration. The specific steps are shown in Figure 16. First of all, the conditions, goals, and needs of the renovation of the public spaces of Tulou buildings should be analyzed to identify contradictory problems through a problem analysis. Secondly, according to the extension transformation of different conditions and goals, multiple different strategies should be formulated. Finally, the most reasonable and feasible renovation plan is selected, and the reuse of the Tulou spaces can be realized [17]. However, in the renovation of micro-space, it is necessary to analyze and renovate based on the actual situation of each Tulou building. For some Tulou buildings, bold attempts can be made by adding or deleting structures such as implanting artistic installations, rather than being limited to traditional renovation thinking. Micro-space re-creation can effectively promote the reuse of Tulou building public spaces and can reduce the waste of resources. Sharing and participating in the micro-space can increase the opportunities and needs of interactions among local residents, can improve relationships among neighbors, can enhance mutual trust and communication, and can increase residents’ sense of identity and belonging to the living environment; thus, achieving the sustainable development of Tulou buildings in the future.

### 4.2. Conclusions

Fujian Tulous are important architectural structures of the Southern Fujian culture and Hakka culture, and they represent the precious heritage of mankind. With the rapid process of urbanization, many Tulou resources are facing the risk of decline due to the lack of awareness regarding preservation and maintenance. In addition, human habits, preferences, and the use and changes in modern spatial planning and development methods have resulted in new requirements for architectural environments, which has resulted in moving a large number of original residents from Tulou buildings, and therefore, the buildings have been gradually become empty. The structure of a Tulou is just a “material organization” or a facade if there are no human beings as well as cultural and social interactions [52]. Therefore, new methods for the renovation and renewal of Tulou buildings are needed to promote their sustainable renewal and to enable residents to continue to live in the cultural and social practice of Tulou architectural heritage sites. The adaptive renovation and renewal of Tulou buildings can reshape a new sense of community, can promote the organic integration of the Hakka culture and modern life concepts, and can meet the living needs of modern people.

Starting from the renovation of Tulou buildings, in this paper, we find that applying extenics theory is a good method to resolve conflicts, which, to a certain extent, can weaken the possibility of conflicts in the renovation of a Tulou, and we explore the feasibility of applying extenics in Tulou renovations. By verifying the application of extenics to the design system for Tulou building renovations, the following conclusions are drawn:

- We combine the theory of extenics with architectural heritage research to build a design system that is suitable for Tulou building renovations. When facing difficult problems in the design of Tulou building renovations, we use the formal expression and paradigm thinking description of extenics to clearly present the design thinking process of Tulou building renovations, which improves the scientificity of design and is conducive to the understanding, acceptance, and dissemination of design thinking.
- Guiding the local residents and other multiple subjects to spontaneously participate in the renovation process can endow local people with creativity, can improve residents’ sense of belonging, can deepen their new appreciation and understanding of local values, and can promote the sustainable renewal of architectural heritage. This bottom-up communication method is more creative than a top-down method, and it has a positive impact on the protection and repair of architectural heritage sites.
- We summarize the relevant suggestions on the regeneration path of Tulou buildings, which can facilitate the renovation and repair of Tulou buildings according to different conditions, and can provide a reference for sustainable development of Tulou buildings in different regions.

The results of this paper show how extenics thinking can be adopted to revive the architectural heritage in ancient villages under physical and regulatory constraints, and to build a design system suitable for the renovation of Tulou buildings, which is conducive to the reuse and redevelopment of other Tulou buildings and architectural heritage sites that are not well known. This innovative renovation can change previous practice, and can provide a practical reference for intangible cultural heritage and other types of architectural heritage buildings. It offers a scientific thinking mode for the future protection and regeneration of architectural heritage sites, promoting sustainable renewal of architectural heritage.

### 4.3. Limitations and Future Research Directions

The content of extenics is rich and this study focuses on the basic element theory and extension transformation method. However, with the development of extenics in architectural design, architectural heritage, etc., and the needs of architectural heritage renovation design practice, different groups have different opinions, which is also a key point to be considered in the design. Extenics is a method to balance the opinions of all parties; extenics can reduce the contradictions to a certain extent, but it cannot completely avoid conflicts. There is still a need to enrich the methodological system of extenics in architectural heritage renovation design research and to strengthen its practical application, so that it can be gradually improved.

Currently, the most significant problem facing the renovation and restoration of traditional buildings is to use design to respond to the contradictions faced by traditional buildings in modern lifestyles, rather than just dealing with the buildings themselves. Thus, here, we integrate a Tulou building renovation with extenics thinking, and use its divergent tree principle to express the complex current conditions of the Tulou building in a symbolic way, in order to understand the Tulou building more comprehensively. Then, we use conjugate pairs to analyze the strengths and weaknesses of the Tulou building, and to find the crux of the contradictory problems of the Tulou building in a short period of time. Finally, with joint participation of users and owners in the design of the Tulou, and according to their actual needs in modern life, we use correlative net, implied system, and split-merge chain analyses to determine an appropriate renovation and repair program, which, to a certain extent, improves the design efficiency. In addition to the renovation of a Tulou, the method of extenics can also be applied to different architectural buildings, such as architectural heritage sites that are more special than the space of a Tulou or ordinary buildings. At the same time, we can analyze extenics thinking and can identify the favorable and unfavorable conditions. In addition, carrying out extension transformation can enhance the practicability of the design scheme and has certain potential reproducibility.

The introduction of extenics in the design process of Tulou renovations is relatively new, with high academic and practical value. However, public participation of different subjects needs to be integrated into the extenics analysis, and, in practice, the renovation design system still needs to be updated and improved. For example, regarding different Tulou renovations, there is still a need to develop different renovation lines in a targeted manner, to strengthen the integration of local culture and to better reduce the contradictions arising from the renovation. Trying to achieve this path is worthwhile, but in the face of different architectural heritage objects, there are certain limitations. For example, regarding objects that have been listed on the World Heritage List, the transformation is more difficult, and it is necessary to analyze the existing conditions layer by layer to ensure the feasibility of the transformation program. Therefore, the renovation design system of this method cannot be applied across the board, but it can be used as a reference. It can be a good reference for traditional and modern buildings that, so far, have not been listed on the World Heritage List; however, for some architectural heritage sites that should clearly not be changed drastically, appropriate adjustments need to be made in a targeted manner to ensure the maximum infusion of vitality while preserving the style.

In this paper, we have only applied basic element theory and its extension transformation method in the design process of Tulou building renovations; we have not carried out in-depth research on other aspects of this theory. Moreover, there is a large number of Tulou buildings in different current situations; thus, in this paper, we have only considered Tulou building renovation projects in Lantian Village, Longyan City as the main research object to verify the feasibility and effectiveness of extenics in the Tulou renovation design process, which leads to certain limitations in this study, and may affect the universality of the verification results. Therefore, it is necessary to verify the renovation of Tulou buildings in other areas to test the applicability of the renovation design system. In other words, we should further study the application of the design system for Tulou building renovations in other areas, and test the replicability of this system. In addition, due to the limitation of the length of this paper, there is no comprehensive discussion of the implementation and application of basic element theory in extenics. In future research, in-depth analysis and discussion will be conducted. The following are suggestions for further research:

- A multi-level analysis and summary of the design of Tulou renovations and other types of architectural heritage sites.
- A systematic analysis of the design system for Tulou renovations to further verify its effectiveness and replicability, avoiding loopholes in the process.
- An in-depth analysis of the quantitative methods in extenics, such as a priority degree evaluation. The combination of qualitative and quantitative methods could make the renovation design more scientific.

## Figures and Tables

**Table 1:** The three basic elements.

Basic Elements	Matter Element, M	Affair Element, A	Relation Element, R
Scope	Contains all subjective and objective existence	Objective natural movements, subjective behavioral activities	The relationships among all things, people and things in nature
Core	Formalizing the noun objects	Formalize the verb objects	Describe the relationship
Goals	A more comprehensive understanding of things	A more comprehensive understanding of the behavioral activities of events	A more comprehensive understanding of the relationships among all things
Features	Emphasis on the subjective and objective description of information about the object of study, more comprehensive and accurate	Describe the laws of events and their behavioral activities	Describe the relationships among all things in the world
Expression form	$M=\left[\begin{array}{r} o_{m},C_{m1},V_{m1}\\ {}_{}C_{m2},V_{m2}\\ \begin{array}{r} \begin{array}{rrr} & \cdots & \cdots \end{array}\\ {}_{}C_{mn},V_{mn} \end{array} \end{array}\right]$	$A=\left[\begin{array}{r} o_{a},C_{a1},V_{a1}\\ {}_{}C_{a2},V_{a2}\\ \begin{array}{r} \begin{array}{rrr} & \cdots & \cdots \end{array}\\ {}_{}C_{an},V_{an} \end{array} \end{array}\right]$	$R=\left[\begin{array}{r} o_{r},C_{r1},V_{r1}\\ {}_{}C_{r2},V_{r2}\\ \begin{array}{r} \begin{array}{rrr} & \cdots & \cdots \end{array}\\ {}_{}C_{rn},V_{rn} \end{array} \end{array}\right]$

**Table 2:** The conjugate pair analysis of a Tulou.

Research Object	Conjugate Pair	Meaning of the Conjugate Part
Tulou	Virtual and real	re: Architectural heritage, cultural objects, etc.
		Im: Spatial scale, etc.
	Soft and hard	sf: Historical culture, folk culture, Hakka culture, spatial functions, etc.
		hr: Tulou buildings, village environment, etc.
	Potential and obvious	it: History and culture that have not been excavated, etc.
		ap: Tulou buildings that have been exploited, etc.
	Positive and negative	psc: Economic benefits brought by tourism, etc.
		ngc: Total consumption value of Tulou building renovation, etc.

## Data Availability

The data used to support the findings of this study are available from the corresponding author upon request.
